# Anticoagulation activity of sulfated carboxymethyl cellulose/*Azadirachta indica* leaf powder-based bio-composite

**DOI:** 10.1039/d4ra02893g

**Published:** 2024-07-12

**Authors:** Khalid A. Alamry, Mahmoud A. Hussein, Ajahar Khan, Abdullah M. Asiri

**Affiliations:** a Faculty of Science, Department of Chemistry, King Abdulaziz University Jeddah 21589 Saudi Arabia kaalamri@kau.edu.sa maabdo@kau.edu.sa; b Chemistry Department, Faculty of Science, Assiut University Assiut 71516 Egypt; c Department of Food and Nutrition, Bionanocomposite Research Center, Kyung Hee University 26 Kyungheedae-ro Dongdaemun-gu Seoul 02447 South Korea; d Centre of Excellence for Advanced Materials Research, King Abdulaziz University Jeddah 21589 Saudi Arabia

## Abstract

Polymeric bio-composites synthesized *via* a green approach using natural herbs have fascinating anticoagulant activity due to their eco-friendly and non-toxic behavior towards various physical and chemical actions. Herein, we introduce a simple and eco-friendly approach for the fabrication of a new hybrid type of bio-composite based on sulfated carboxymethyl cellulose (S-CMC) and *Azadirachta indica* leaf powder (S-CMC/NLP). First, a non-toxic sulfating agent called N(SO_3_Na)_3_ was used to modify carboxymethyl cellulose into S-CMC. With an ion exchange capacity of 0.25 meq. g^−1^, the level of sulfation (%) of S-CMC (modified polysaccharide) was measured to be 12.01%. Three types of S-CMC/NLP bio-composites were developed by varying the concentration of NLP. FE-SEM, EDX, and XRD were used to characterize the structural features of S-CMC/NLP bio-composites. FTIR spectroscopy indicated that the S-CMC/NLP bio-composite possesses COO^−^, –OH and SO^3−^ groups, suggesting the structural similarity to heparin. In addition, the anticoagulant effect of the S-CMC/NLP bio-composite was investigated using PT and APTT assays. The APTT investigation confirmed that following the intrinsic pathway of the coagulation system, 2-NLP/S-CMC bio-composite dose-dependently (0.045–0.28 mg mL^−1^) prolonged the time of blood coagulation compared to control (pure plasma). The S-CMC/NLP bio-composite showed its potential as a new, safe, and effective candidate for anticoagulant activity.

## Abstract 2

The eco-friendly and non-toxic behavior of polymeric bio-composites based on S-CMC, synthesized utilizing natural herbs (*Azadirachta indica* leaf powder), has shown an interesting anticoagulant activity against diverse physical and chemical actions.

## Graphical abstract

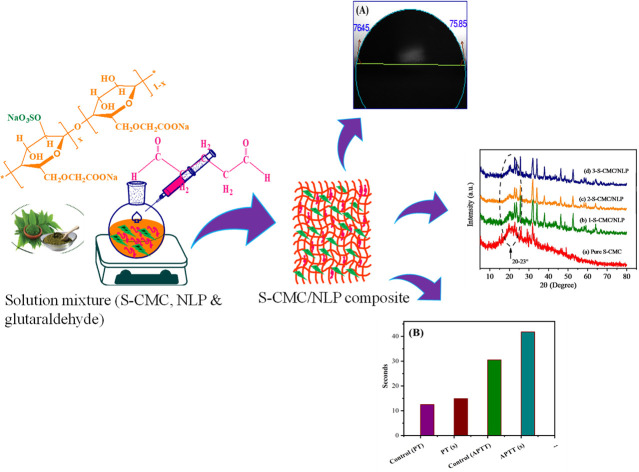

## 1. Introduction

Currently, with the advancement of technology in the field of clinical science, various types of biomaterials such as ceramics, metallic components, composite materials and natural or synthetic polymers have been employed for a variety of medical applications.^1–4^ Over time, these biomaterials have expanded in order to fulfill the demand of clinical needs both in therapeutic as well as diagnostic fronts.^5^ The characteristic features of natural and synthetic materials used in clinical plots, where they directly make contact with blood, are essential to the obtained results of graft or implant. Physiologically adequate biomaterial/blood agreement is vital for the flourishing blood-contacting biomaterial applications. At the time of blood clotting, plasma proteins (primarily fibrinogen, globulin, serum albumin and prothrombin) are hastily absorbed over the surface of the material in the presence of a foreign body, which stimulates the platelets and persuades their aggregation and adhesion for blood clotting.^6^ Heparin is a naturally acquiring anticoagulant, which is most widely used for this aspect, made up of sulfated polysaccharide backbones constituting repeating units of d-glucosamine and either d-glucuronic or l-iduronic acids.^7^ The presence of carboxylate, sulfamide and sulfate-based ionic functional groups are key factors of heparin for anticoagulant activity, which helps to immobilize heparin onto the material to improve the blood compatibility *via* ionic bonding or physical absorption.^8–11^ For many decades, heparin has been found to be a main choice for the treatment and prevention of thromboembolic disorders. However, some problems related to its clinical applications have also been reported; for instance, in its inadequacy for antithrombin deficient patients, the immobilized heparin straightforwardly leaves the material surface and comes into the blood, which results in the abnormal flow of blood and can lead to consequent tissue hemorrhage and heparin convinced thrombocytopenia because of serious side effects.^12,13^ Moreover, the literature review suggested that sulfated polysaccharides, including natural or chemically synthesized ones, also possess immense anticoagulant activity and blood compatibility.^14,15^ Cellulose derivatives such as carboxymethyl cellulose (CMC) can be obtained in large quantities through the treatment of cellulose with sodium mono chloroacetate in the presence of sodium hydroxide, attributing to a variety of applications including detergents, papers, flocculation, textiles, foods and drug release.^16,17^ The large proficiency of carboxy methyl groups (–CH_2_COO^−^) in the CMC evidently enhances the water solubility.^18,19^ Moreover, after sulfation, CMC also possesses significant blood compatibility because of the similarity in the structure with that of heparin. Among different types of biomaterials, plant extracts have achieved modest consideration owing to their easy availability, low cost and eco-friendly nature. *Azadirachta indica* (*A. indica*), which belongs to the family Meliaceae, is an evergreen and indigenous plant widely available in India and southeast countries. This plant has promising potential for ayurvedic therapeutics owing to its remedial properties with proven anti-inflammatory, antiviral, antidiabetic, antifungal, skin healing, anti-ulcer and antiretroviral activities.^20–22^ Moreover, the water-soluble leaf extract of *A. indica* also possesses antifertility, anti-serotonin, hypotensive, hypoglycemic, and hepatoprotective activities.^23^ Over time, researchers have investigated the fact that *Azadirachta indica* is loaded with several types of compounds, of which a wide range of compounds have pharmacological potential. Triterpenes lead the way in having a therapeutic application, and Nimbin (triterpene) has been shown to have antiseptic, antihistamine, fungicidal and antipyretic properties. Nimbin also exhibits antioxidant and anti-inflammatory activities, therefore dropping damage by mitigating the formation of oxygen reactive species.^24,25^ Flavonoids are also found in neem, which works as inhibitors of prostaglandin biosynthesis, phosphodiesterases, endoperoxides and enzymes like protein kinases, all concerned with inflammation.^25–27^ On the other hand, oil extracts of *Azadirachta indica* are the most distinctive used form, and its phytochemical investigation has revealed the occurrence in large amounts of saponins, flavonoids, and triterpenes, while other components, for instance, nimbins and catechins appear to occur in lower amounts.^24,25^ Other metabolites present in *A. indica* extracts are alkaloids, limonoids, tannins, gallic acid, sterols, catechins, terpenoids and reducing sugar.^24,25,28,29^ The leaf of *A. indica* seems to have developed a specific set of glycoproteins that, when analyzed on mammalians, confirmed immune–modulatory activity, inhibiting the growth of tumors by modulating systemic and local immunity.^30–33^ A literature survey reported that leaf extracts of *A. indica* indicate high levels of glycosides, tannins, saponins, flavonoids, and alkaloids.^34^ The potential effects that are seen when using *A. indica* extracts can positively be attributed to cellular and molecular mechanisms, which comprise detoxification, free radical scavenging, DNA repair, immune surveillance, programmed autophagy and cell death mitigation, cell cycle alteration, anti-inflammatory, anti-metastatic and anti-angiogenic activities and the capability to modulate different signaling pathways.^35,36^ The anti-inflammatory activity of NLP is due to the presence of the bioactive compound limonoid.^37,38^

This research article addressed the fabrication of cost-effective and eco-friendly bio-composites based on sulfated-carboxymethyl cellulose (S-CMC) and the *A. indica* leaves (NLP) through a simple and green chemical approach. To demonstrate the influence of *A. indica* leaves in the fabricated S-CMC/NLP bio-composites, anticoagulant activities of fabricated S-CMC/NLP bio-composites were investigated. The modified sodium carboxymethyl cellulose sulfates were prepared with a special sulfating agent (N(SO_3_Na)_3_), which was developed in the lab using sodium nitrite and sodium bisulfite in aqueous media. Conventionally, sulfation was carried out by using sulfuryl chloride, sulfuric acid, sulfur trioxide, sulfamic acid and chlorosulfonic acid. These sulfating agents not only result in the intense degradation of polysaccharide polymer backbones but also spark severe pollution problems.^39^ Compared with conventional techniques, the whole fabrication experiment was accomplished in aqueous media.

Moreover, the prepared sulfating agent utilized for the sulfation of CMC was cost-effective and non-toxic. Reactive oxygen and free radical species of *A. indica* are the main sources of inflammation, as they proceed upon different biological molecules, exerting injuries by taking out electrons as a way of inflowing a stable state, unleashing in the cell a condition of oxidative stress.^40,41^ Thus, there is a demand for adequate compounds by compositing with a suitable polymer or filler in order to neutralize or stabilize these radicals as a step in blocking or preventing an exacerbation of oxidative stress. Therefore, considering the importance of green processing and taking into account the significance of S-CMC and the medicinal benefits of NLP, this work proposed the successful fabrication of S-CMC/NLP bio-composites towards anticoagulant potential. Furthermore, to support the experimental procedure, the fabricated S-CMC/NLP-based bio-composites were examined by X-ray diffraction (XRD), FT-IR, FE-SEM and EDAX. Moreover, the fabricated S-CMC/NLP bio-composite samples were also analyzed to investigate their anticoagulant effect. The results obtained through partial thromboplastin time (PT) and activated partial thromboplastin time (APTT) assay suggested that the newly fabricated S-CMC/NLP-based bio-composites may be considered potential candidates for chemicals pharmaceutical and clinical applications.

## 2. Experimental

### 2.1. Materials and methods

Sodium carboxymethyl cellulose sodium salt (having low viscosity, the viscosity of a 1% solution in water at 20 °C 30–70 c/s. pH of 2% solution 6–8) was purchased from BDH Chemicals Pvt. Ltd Poole, England. Sodium nitrite and sodium bisulfite were procured from Fisher Scientific Chemicals Pvt. Ltd. Sodium hydroxide, ethanol, and acetone were purchased from Sigma-Aldrich (USA), *Azadirachta indica* (neem) leaves were collected from Jeddah, Saudia Arabia, and other reagents were of analytical grade and utilized without additional purification.

### 2.2. Accumulation and preparation of *A. indica* leaf (NLP) powder

Fresh leaves of *A. indica* (neem) were collected from Jeddah, Saudia Arabia. The fresh young leaves, after separating from twigs, were washed with tap water thoroughly four times and then treated with double distilled water (DDW) three times, followed by drying in a hot air oven at 40 °C for 72 h. The dried leaves were ground into a fine powder using a mortar and pestle. The obtained powder was washed again with acetone and DDW to remove any dirt or impurity remains, followed by drying at 37 °C. Finally, the obtained dried leaf powder was composited with S-CMC at varying concentrations.

### 2.3. Fabrication of sulfated-CMC and biocomposite

**Fig. 1:**
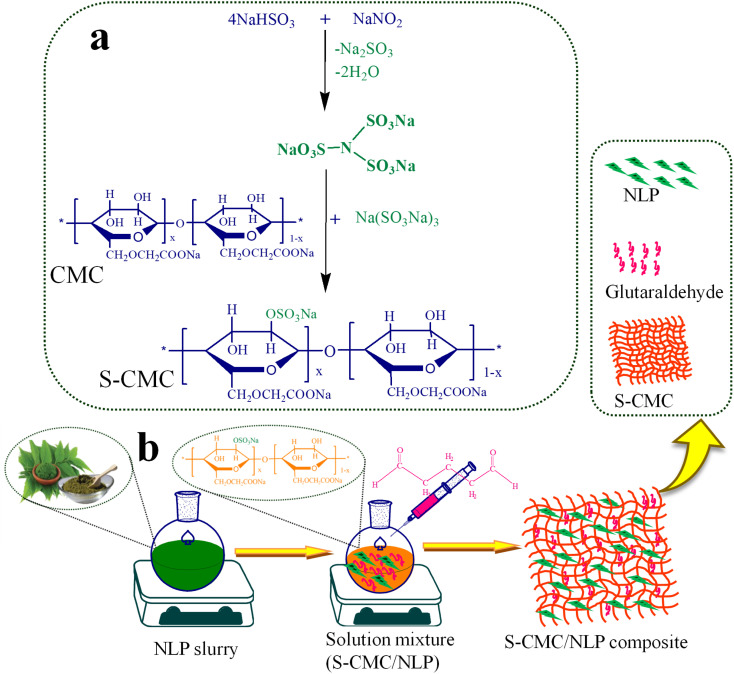
(a) Plausible mechanism for the synthesis of sulfated carboxymethyl cellulose (S-CMC) and (b) schematic representation of the fabrication of S-CMC and *Azadirachta indica* leaf powder (S-CMC/NLP) bio-composites.

The synthesis of sulfated-CMC (S-CMC) was performed as given in the previous literature with slight modification.^42^ First of all, a special sulfating agent was prepared using sodium nitrite and sodium bisulfate. Typically, 0.1 M sodium bisulfate was dissolved in 80 mL DDW in a round bottom flask fitted with a condenser. After that, 1 M sodium nitrite prepared in 20 mL DDW was carefully dropwise added into the round bottom flask under constant mechanical agitation at 90 °C up to 1.5 h to form (N(SO_3_Na)_3_). Finally, the pH of the prepared sulfating agent was adjusted to ∼8 using 0.2 M NaOH. Now, 10 g of CMC was consequently added slowly to the prepared solution under strong mechanical agitation (600 rpm) and allowed the reaction to proceed for 5 h at 45 °C. The highly swollen gel suspension formed was dried at 50 °C to evaporate the solvents completely. Afterward, the obtained dried S-CMC was thoroughly washed with DDW and acetone, followed by complete drying at 60 °C (Fig. 1(a)).

In the next step, the S-CMC/NLP bio-composites were fabricated through a simple solution casting method. In detail, the different concentrations of *A. indica* leaf powder (NLP), 2.5%, 5.0% and 7.5% (w/v), were poured into 3 different round bottom flasks containing 20 mL double distilled water, each under constant stirring for 5 h at 45 °C, followed by sonication up to 1 h. Now, 5.0% of dried S-CMC was added subsequently into all the three above solution mixtures under constant stirring at 45 °C. After adding 2 mL of glutaraldehyde (as crosslinker) into each solution mixture, the reactions proceed under strong mechanical agitation for 36 h at 45 °C, followed by sonication for 3.5 h. Then, the obtained S-CMC/NLP biocomposite mixtures were cast into Petri dishes and kept in a hot air oven at 60 °C to completely dry (Fig. 1(b)). Finally, the dried S-CMC/NLP composites were treated with acetone for 2 h and crushed into a fine powder using mortar and pestle for further characterization. Herein, on the basis of varying concentrations of NLP, *i.e.*, 2.5%, 5.0% and 7.5%, the synthesized S-CMC/NLP bio-composites were named as 1-S-CMC/NLP, 2- S-CMC/NLP and 3- S-CMC/NLP respectively.

### 2.4. Ion exchange capacity and level of sulfation

The level of sulfation (%), which represents the average number of sulfonic (–SO_3_^−^) groups per repeat unit in the polymer backbone, can be used to investigate the ion exchange capacity (meq. g^−1^). The ion exchange capacity of S-CMC was calculated through a classical titration method.^43^ The level of sulfation (%) of S-CMC was carried out using a slightly modified approach, as mentioned by Unveren *et al.*^44,45^ Typically, ion exchange capacity measures H^+^ ions released by neutral salts to move through the polymer backbone. In brief, the dried S-CMC (0.25 g) was immersed in the aqueous solution of 1 M HNO_3_ for 24 h to convert into the H^+^ form. Then, S-CMC was washed with double distilled water till neutralization, followed by complete drying at 60 °C. After that, the dried S-CMC was packed into a glass column. After that, 1 M NaNO_3_ as eluent was used to elute the protons totally from the column, with a flow rate of 0.4 mL min^−1^. Then, the exchanged proton (H^+^) of the solution was titrated using phenolphthalein as an indicator with standard (0.1 M) NaOH solution. The ion exchange capacity and level of sulfation (%) of S-CMC were investigated *via* the following equations:1
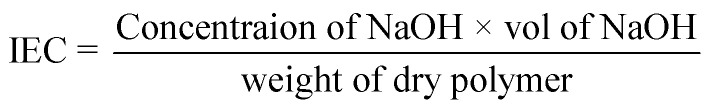
2
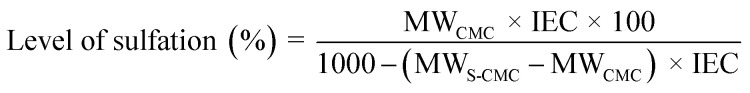
MW_CMC_ and MW_S-CMC_ are the molecular weights of two monomer units of CMC (468.176) and S-CMC (570.233), respectively.

### 2.5. Characterization

The elemental composition of S-CMC/NLP biocomposites was measured by energy dispersive X-ray (EDX) (Oxford instruments INCA, X. act, S. No. 56756, UK). Structural description and morphological appearance were examined by field emission scanning electron microscopy (FE-SEM) at a Zeiss Evo 50 XVP, UK. The structural pattern of fabricated biocomposites was determined by X-ray diffraction (XRD) pattern using Rigaku, Miniflex-II-Japan with Cu Kα (at 40 kV, 40 mA, and 2*θ* with a scan angle: 10°–80°). The chemical structure was characterized with Fourier transform infrared spectra (PerkinElmer 100 FT-IR Spectrometer-USA) by recording in the range of 500 to 4000 cm^−1^. The OCA 15 Pro commercial goniometer (Dataphysics, Germany) was used to measure the contact angle. The instrument was equipped with a stepper motor for controlling the amount of water dispensed from a micro syringe and a CCD camera attached to the instrument that operates in static mode to capture digital images of the water droplets on the surface. All the S-CMC/NLP samples were completely dried at 45 °C and packed in airtight culture tubes prior to characterizations. The anticoagulant properties of S-CMC/NLP biocomposites were recorded using PT and APTT assay.

## 3. Results and discussion

The present work aimed to discourse the potential evaluation of anticoagulation properties of NLP-supplemented S-CMC-based biocomposite. For this, a simple and facile route was adopted to fabricate “green, cost-effective and eco-friendly S-CMC/NLP bio-composites. Herein, the synthesized S-CMC/NLP bio-composites were named 1-S-CMC/NLP, 2- S-CMC/NLP and 3- S-CMC/NLP. The anticoagulant properties of polysaccharides are prejudiced by structure and the presence of sulfonated groups.^46^ Therefore, the modification of CMC into S-CMC was carried out to introduce sulfonic groups into the polymer backbone. To substitute the traditional methods, a new type of low-cost, non-toxic, sulfating agent (N(SO_3_Na)_3_) was used to get S-CMC that permits the whole modification reaction to be carried out in an aqueous solution. The ion exchange capacity value of the S-CMC polysaccharide was found to be 0.25 meq. g^−1^. From the experimental value of ion exchange capacity, the level of sulfation (%) of the modified polysaccharide (S-CMC) was calculated to be 12.01%. NLP has active compounds that have antibacterial, antifungal and antiviral properties and is considered a promising natural herb owing to its probable broad-spectrum therapeutic and prophylactic role due to the presence of bioactive compounds.^47–51^ These bioactive compounds may also be able to alter the serum biochemical and hematological parameters of animals.

Furthermore, the favorable effects of *A. indica* have been studied, which is found to have anti-oxidative activity, anti-inflammatory, cancer chemo-preventive potential, hepato-protective, and be effective as an anti-diabetic agent.^52–57^ The plausible interactions between S-CMC and NLP during the compositing process might involve hydrogen bonding among H atoms of NLP (from amino and hydroxyl groups) and sulfonic groups of S-CMC. The formation of hydrogen bonding can be confirmed by the shifting of the –O–H stretching wavenumber from ∼3400 to 3350 cm^−1^. Moreover, the fabricated S-CMC/NLP bio-composite possesses anticoagulant properties due to a large profusion of hydroxyl (–OH), sulfonyl (–SO^3−^) and carboxylate (–COO^−^) functional groups on the S-CMC chain, which helps the polymer to get interactions with specific proteins, especially antithrombin, like heparin polymer.^58,59^ Finally, for the prepared S-CMC/NLP bio-composite, the experimental outcomes indicated that the modification of CMC followed by compositing with NLP enhanced its anticoagulant activity, which may help promote the wound healing process.

### 3.1. FT-IR analyses

**Fig. 2:**
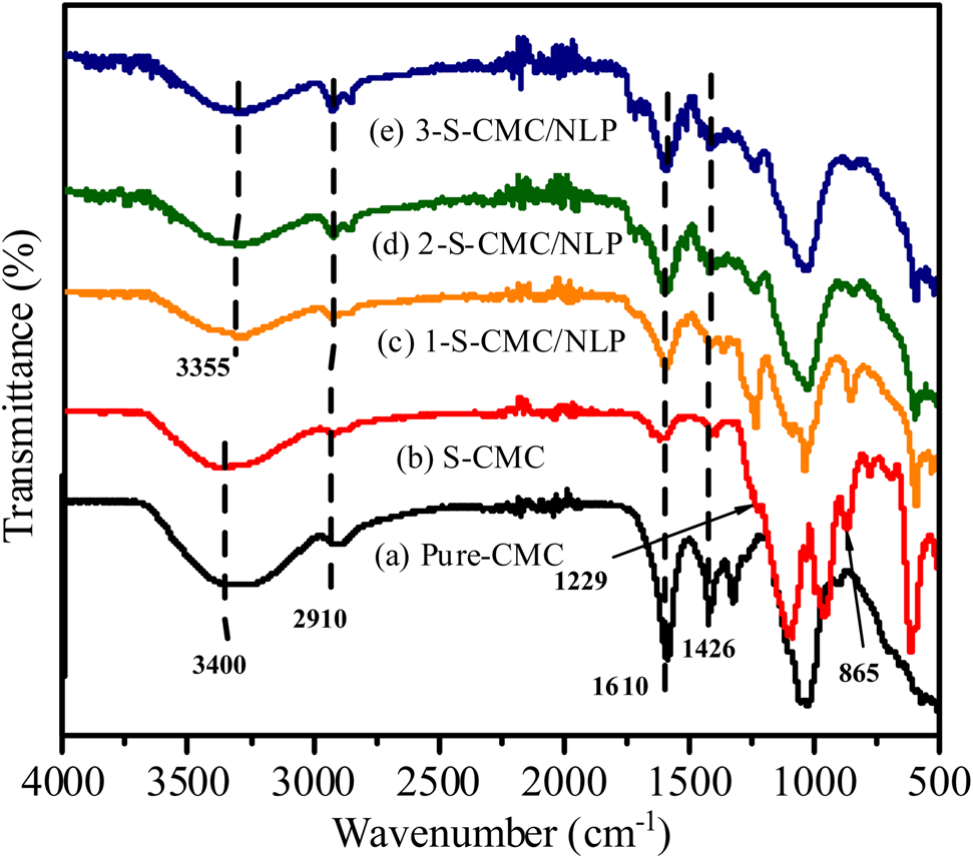
FTIR spectra of (a) pure carboxymethyl cellulose (CMC), (b) sulfated carboxymethyl cellulose (S-CMC), (c) S-CMC and 0.5 g *Azadirachta indica* leaf powder (1-S-CMC/NLP), (d) S-CMC and 1.0 g *Azadirachta indica* leaf powder (2-S-CMC/NLP), and (e) S-CMC and 1.5 g *Azadirachta indica* leaf powder (3-S-CMC/NLP) bio-composites.

The FT-IR spectra of Pure CMC, S-CMC and 1-S-CMC/NLP, 2-S-CMC/NLP and 3-S-CMC/NLP bio-composite are shown in Fig. 2. Typical absorption bands of fabricated bio-composites at ∼3400, 2910, 1610, 1426 and 1054 cm^−1^ were clear for all five samples. These characteristic absorption bands correspond to the –O–H and –C–H stretching vibrations, –C=O vibrations and carbonyl –C–O stretching vibrations, respectively, of CMC, S-CMC and S-CMC/NLP bio-composite.^60^ Moreover, it can be observed from Fig. 2(b) that the S-CMC consists of two characteristic absorption bands; one at 1229 cm^−1^ was ascribed to the asymmetrical stretching vibration of –S=O and the other absorption band at 865 cm^−1^ was attributed to the symmetrical stretching vibration of C–O–S associated with the C–O–SO_3_ group.^61^ However, these two characteristic absorption bands were absent in the FTIR spectrum of pure CMC (Fig. 2(a)), which reveals that the sulfation of CMC was successfully achieved with the introduction of the sulfate group into the polymer chain of S-CMC. On the other hand, due to hydrogen bonding interaction between hydrogen atoms of NLP (from amino and hydroxyl groups) and sulfonyl group of S-CMC, the absorption band due to –O–H stretching shifts from ∼3400 to 3350 cm^−1^ (Fig. 2 (c)–(e)). It has to be noted that the intensity of characteristic absorption bands ascribed for –OSO_3_ groups in S-CMC diminished in 1-, 2- and 3-S-CMC/NLP biocomposites with marginal shift from 870 cm^−1^ to 854 (Fig. 2(c)–(e)), which is in good agreement, confirming the successful interaction among the active sites present within the developed bio-composite.

### 3.2. FE-SEM analysis

**Fig. 3:**
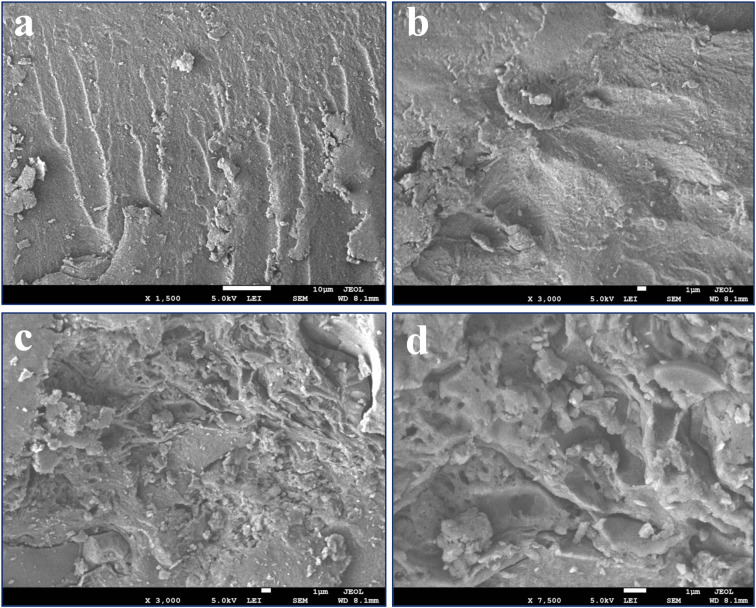
SEM images of sulfated carboxymethyl cellulose and *Azadirachta indica* leaf powder (S-CMC/NLP) bio-composite (a) and (b) SEM image of pure S-CMC, and (c) and (d) SEM images of S-CMC supplemented NLP based bio-composite scanned at different magnifications.

FE-SEM micrographs were obtained to observe the shape and surface morphology of the fabricated S-CMC/NLP bio-composite. Fig. 3(a)–(d) shows FE-SEM images of pure S-CMC and S-CMC/NLP biocomposites at low (left) and high magnifications (right). The surface of the pure S-CMC biomaterial has compact, homogeneous structural integrity with low roughness on the surface (Fig. 3(a) and (b)). S-CMC morphology alone was relatively more rigid (Fig. 3(a) and (b)), which could be due to more intensive attraction among the polymer backbone after sulfation. On the other hand, by compositing S-CMC with NLP, the whole surface becomes more porous, rougher and hills with enhanced NLP and reduced S-CMC amounts (Fig. 3(c) and (d)). In addition, spherical or differently shaped aggregates were observed on the surface of the S-CMC/NLP bio-composites (Fig. 3(c)). Further magnification (Fig. 3(d)) suggests the presence of the granular aggregates strongly bound, engulfed, and uniformly dispersed on the surface of the bio-composite as a wall-forming material. These observations confirm that a well-defined matrix was formed by the composition of S-CMC and NLP biomaterials.

### 3.3. EDX analysis

**Fig. 4:**
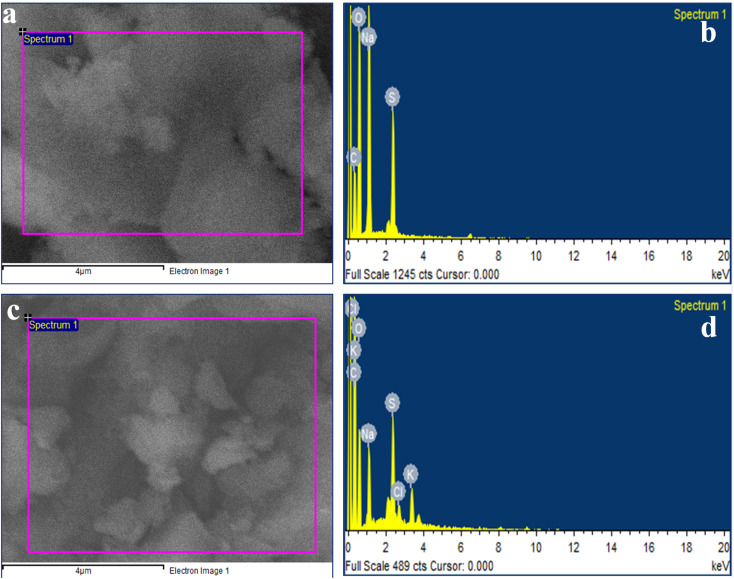
EDAX analysis of (a) and (b) sulfated carboxymethyls cellulose (S-CMC) and (c) and (d) S-CMC and *Azadirachta indica* leaf powder (S-CMC/NLP) bio-composite fabricated through green chemical approach.

EDX analysis was carried out to confirm the sulfonation of CMC and the composition of S-CMC/NLP-based bio-composites (Fig. 4). For pure S-CMC biomaterial (Fig. 4(a) and (b)), the presence of sharp elemental peaks of S along C, O and Na confirm the sulfonation of CMC biopolymer. The presence of S can also be observed in the composition of both pure S-CMC and S-CMC/NLP, which suggests the successful modification of sodium carboxymethyl cellulose into S-CMC (Fig. 4(b) and (d)). Furthermore, in the composition of S-CMC/NLP, the additional elemental peaks of K and Cl, along with C, O, S and Na, can be clearly observed (Fig. 4(d)). Other elemental peaks observed in both EDX spectra were due to the presence of various functional groups in the S-CMC chain.

### 3.4. XRD analysis

**Fig. 5:**
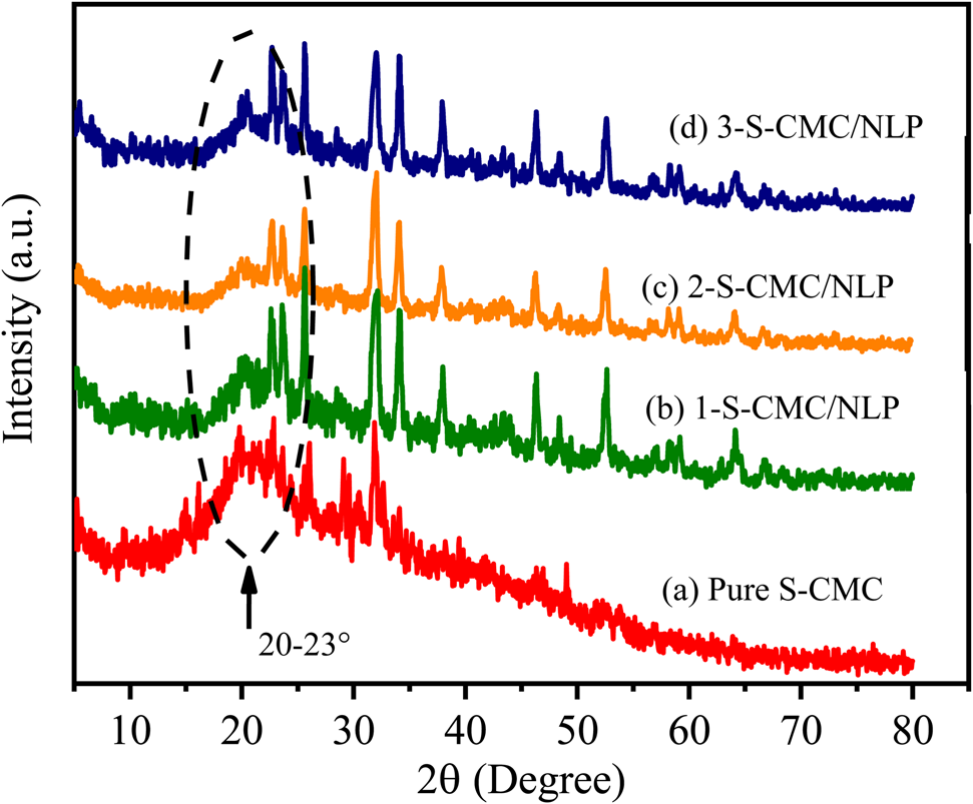
XRD diffraction patterns of (a) sulfated carboxymethyl cellulose (S-CMC), (b) S-CMC and 0.5 g *Azadirachta indica* leaf powder (1-S-CMC/NLP), (c) S-CMC and 1.0 g *Azadirachta indica* leaf powder (2-S-CMC/NLP), and (d) S-CMC and 1.5 g *Azadirachta indica* leaf powder (3-S-CMC/NLP) bio-composites.

The XRD patterns of fabricated S-CMC, 1-, 2- and 3-S-CMC/NLP biocomposites are shown in Fig. 5. It can be observed from Fig. 5(a) that sulfated-CMC represent typically non-crystalline morphology with a wide diffraction peak in the region of 2*θ* = 20–23°, which can be assigned due to the reflection of the (200) plane and confirms the amorphous nature (Fig. 5(a)).^62^ This similar broad peak (2*θ* = 20–23°) can also be seen in the XRD pattern of the 1-, 2- and 3-S-CMC/NLP biocomposites (Fig. 5(b)–(d)), which indicates the successful composition of NLP with S-CMC without impairing the nature of modified S-CMC. However, after compositing of S-CMC with NLP, the intensity of the diffraction peaks increases due to the presence of different metal elements in NLP (Fig. 5(c) and (d) ); therefore, the XRD pattern of the fabricated biocomposites exhibits small peaks of 2*θ* values (Fig. 5(b)–(d)). The increasing intensity of diffraction peaks with the existence of broad diffraction peaks (2*θ* = 20–23°) indicates an amorphous nature with the semi-crystalline structure of the fabricated S-CMC/NLP-based biocomposites.

### 3.5. Anticoagulant activity of S-CMC/NLP-based biocomposite

**Fig. 6:**
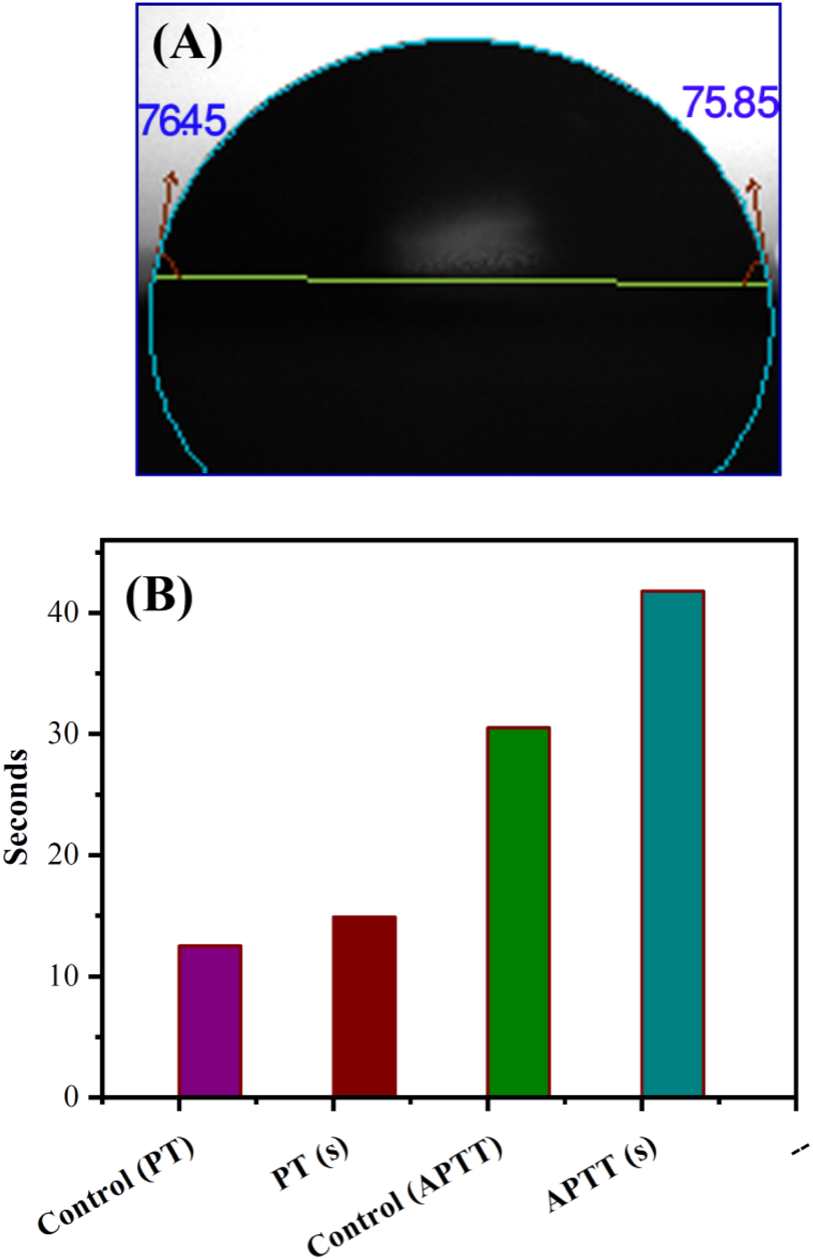
(A) Image of contact angle in the form of a water droplet for sulfated carboxymethyl cellulose/*Azadirachta indica* leaf powder (S-CMC/NLP) bio-composite. (B) Coagulation time in seconds activated partial thromboplastin time (APTT) and prothrombin time (PT) levels for control compared to the fabricated S-CMC/NLP bio-composite PT(s) and APTT(s) represent biocomposite S-CMC/NLP.

**Fig. 7:**
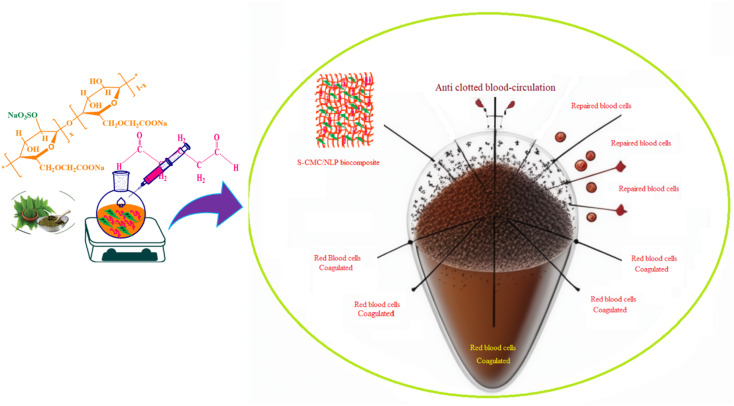
The proposed anticoagulation activity with a schematic representation of the designed S-CMC/NLP biocomposite.

The hydrophilicity of the used composite materials has affirmative impacts on anticoagulant activities, and the contact angle measurement is an important parameter for investigating the hydrophilicity of that composite. The contact angle determination can be achieved by the assessment of a water droplet on the surface of a biocomposite material. The low value of the contact angle demonstrates the high hydrophilic nature of the tested material. As shown in Fig. 6(A), S-CMC/NLP-based biocomposite showed a value of 76.45°(left)/75.85°(right). This lower value of contact angle confirms the hydrophilic nature of the proposed S-CMC/NLP biocomposite. The suitable hydrophilicity of a composite surface can suppress the adhesion of platelets to the used composite material, which is essential for blood compatibility.^63^ Platelets are negatively charged because of the occurrence of a negatively charged sugar sialic acid on their surfaces.^64^ The negatively charged sulfonyl (–SO^3−^) and carboxylate (–COO^−^) groups of S-CMC/NLP-based biocomposite are electro-statically repelling toward the side of platelets, which hinder the absorption of platelets on the S-CMC/NLP surface.^63^ Thus, the hydrophilic nature of S-CMC/NLP bio-composites is due to sulphonyl (–SO^3−^) and carboxylate (–COO^−^) groups, which obstruct platelet adhesion on the material surface, further establishing robust anticoagulant activities of the proposed bio-composite. Low platelet adhesion and activation demonstrate enhanced blood compatibility. The activation of blood plasma proteins induces blood clotting at the time of their adsorption on an external surface. The activation of coagulation factors caused the transformation of prothrombin into thrombin, which induced the transformation of fibrinogen into fibrin protein and produced blood clot.^65^ The two main routes to activate plasma proteins that induced blood coagulation are: the extrinsic and intrinsic pathways.^66^ Therefore, the time required for blood clotting has been considered as a critical factor to appraise any external surface in order to establish its antithrombogenicity. Partial thromboplastin time (PT) and activated partial thromboplastin time (APTT) are two factors used to divulge the clotting variations that are concerned with extrinsic and intrinsic pathways and also considered as good indicators to determine the clotting time.^67^Fig. 6(B) shows the anticoagulant activity of the fabricated S-CMC/NLP-based biocomposite with respect to the control PT and APTT values. The coagulation factors measured by APTT assay follow an intrinsic pathway, while after the addition of plasma sample, TT assay investigates the formation time of fibrin from fibrinogen.^68^ As can be observed from Fig. 6(B), PT and APTT levels for 2-S-CMC/NLP (PT(s)) and (APTT(s)) biocomposite were prolonged in comparison with control (pure plasma). It seems the proposed biocomposite possessed high PT and APTT levels in comparison to the control one, so the composition of NLP with S-CMC provided better anticoagulant activities than pure plasma. The investigation of the intrinsic pathway of the coagulation system through the APTT evaluation confirmed that the 2-NLP/S-CMC bio-composite dose-dependently (0.045–0.28 mg mL^−1^) prolonged the time of blood coagulation compared to control (pure plasma). The coagulation time was predicted by the varying concentrations of biocomposites (1-S-CMC/NLP, 2-S-CMC/NLP, and 3-S-CMC/NLP). Furthermore, 2-NLP/S-CMC bio-composite increased the coagulation time at 0.28 mg mL^−1^, being better than the 1-NLP/S-CMC and 3-NLP/S-CMC) biocomposites, and much less than the effect demonstrated for heparin, requiring a larger amount of 2-NLP/S-CMC biocomposite in order to attain a similar anticoagulant activity. Herein, the anti-inflammatory activity of NLP is due to the presence of the bioactive compound limonoid.^37,38^ Limonoid is a furanolactone, identified for its inhibitory effects in the fabrication of inflammatory mediators. It is also considered a pain anesthetizer as it promotes the activation of endogenous opioid pathways.^24,25,38^ However, these herbs in their natural form allow limited acceptability in the potential application, which has been overcome by formulating a new bio-composite by using NLP with S-CMC in the form of suitable products to serve as an effective anticoagulant material. Furthermore, polyanionic S-CMC could interact with cationic groups present on the cell surface, thereby enhancing membrane permeability, which in turn leads to leakage and disruption of cellular proteins. This electrostatic interaction facilitates the higher activity of S-CMC against different Gram-negative and Gram-positive bacteria, which can be further enhanced by adding *A. indica* extract (NLP).^69,70^ On the other side, the high degree of sulfation and concentration could enhance the density negatively to inhibit the activity of thrombin IIa factor and Xa factor.^71^ Therefore, the sulfated form of CMC inhibited thrombin Xa factor and IIa factor to produce anticoagulant activity. APTT assay is an essential indicator of coagulation activity. The APTT value suggested that the anticoagulant activity of S-CMC/NLP-based biocomposite was good. Therefore, it can be assumed that the sulfate group plays a significant role in enhancing the anticoagulant activity. Similar to heparin, S-CMC into S-CMC/NLP-based bio-composite with the sulfate groups (negatively charged) neutralizes the positively charged residues of amino acids present in the anti-thrombin in order to enhance anticoagulant activities. From this analysis, we speculated that the fabricated S-CMC/NLP bio-composite possesses anticoagulant activities due to a large profusion of hydroxyl (–OH), sulfonyl (–SO^3−^) and carboxylate (–COO^−^) functional groups on the S-CMC chain, which helps the biocomposite to interact with the active site of specific proteins, especially antithrombin, like heparin polymer.^58,59^ The negatively charged sulfonyl (–SO^3−^) and carboxylate (–COO^−^) reduce the protein adsorption through the electrostatic effect, which drops the activation of intrinsic cascade.^65^ This negatively charged surface is accessible for binding coagulation factors, which results in long APTT.^72^ The coagulation flow initiates platelet activation and aggregation and induces thrombosis and subsequent coagulation.^73^ Therefore, this research has shown that the combination of NLP of *A. indica* and S-CMC synergistically into the proposed bio-composite stimulates anticoagulant activity through improved inflammatory response and interactions with the active site of specific proteins. Moreover, S-CMC and NLP are both biodegradable and nontoxic towards mammalian cells. An illustration of anticoagulation activity with a schematic representation of the designed S-CMC/NLP biocomposite has been displayed, as shown in Fig. 7. Furthermore, a comparison between the proposed biocomposite and others reported previously has been summarized in Table 1.

**Table 1:** Comparison of clotting times investigated for S-CMC/NLP biocomposite with respect to the other reported biomaterials

S. no.	Materials	PT (second)	APTT (second)	Ref.
1	Sturgeon skull chondroitin sulfates	10.60	34.60	74
2	Polyethersulfone/4-amino benzene sulfonamide	13.03	37.51	75
3	Polyethersulfone/4-amino-*N*-(5-methyl isoxazol-3-yl) benzene sulfonamide	12.23	34.01	75
4	Cellulose sulfate sodium	11.60	33.20	76
5	Heparin	12.20	39.50	76
6	Sulfated polysaccharide (*Gelidiella acerosa*)	—	37.20	77
7	S-CMC/NLP biocomposite	15.08	41.96	Our work
8	Control	12.54	30.56	Our work

## 4. Conclusion

In the current work, a cost-effective and biocompatible biocomposite was successfully prepared from sulfated-CMC and NLP (S-CMC/NLP) through a simple and easy approach in an aqueous medium without using any toxic reagent. The modification of CMC was carried out in an aqueous medium using a neutral N(SO_3_Na)_3_ sulfating agent. Subsequently, S-CMC was combined with NLP to create a biocomposite. The composition and structure of the proposed biocomposites were characterized by FT-IR, FE-SEM, EDX and XRD studies. FT-IR analysis exhibits the characteristic absorption bands at 1229 and 865 cm^−1^ assigned to sulfate ester bonds, which confirms the introduction of sulfate groups. The interactions between S-CMC and NLP during the compositing process are caused by hydrogen bonding that can be confirmed by the shifting of –O–H stretching wavenumber from ∼3400 to 3350 cm^−1^. The XRD pattern showed that S-CMC/NLP corresponded to the semi-crystalline structure without impairing the nature of S-CMC. The anticoagulant properties of S-CMC/NLP biocomposites were measured by PT and APTT assays. The obtained results revealed that the proposed biocomposite exhibited a high anticoagulant activity. According to the Pt and APTT experiments, the S-CMC/NLP had an effective anticoagulant effect owing to the coexistence of –COO^−^, –SO^3−^, and –OH on the surface of the biocomposite.

## Abbreviation

CMC: Pure carboxymethyl cellulose
S-CMC: Sulfated carboxymethyl cellulose
NLP: *Azadirachta indica* leaf powder
S-CMC/NLP: Sulfated carboxymethyl cellulose/*Azadirachta indica* leaf powder
1-S-CMC/NLP: S-CMC and 0.5 g *Azadirachta indica* leaf powder
2-S-CMC/NLP: S-CMC and 1.0 g *Azadirachta indica* leaf powder
3-S-CMC/NLP: S-CMC and 1.5 g *Azadirachta indica* leaf powder
APTT: Activated partial thromboplastin time
PT: Prothrombin time

## Conflicts of interest

The authors reported no potential conflict of interest.
